# Supplementary material for: Maternal Resveratrol Supplementation Attenuates Prenatal Stress Impacts on Anxiety- and Depressive-like Behaviors by Regulating Bdnf Transcripts Expression in the Brains of Adult Male Offspring Rats
Source: Brain Sci. 2025 Feb 19;15(2):210. doi: 10.3390/brainsci15020210 (PMC11853727; doi:10.3390/brainsci15020210)
Supplement: Supplementary file 1 [file brainsci-15-00210-s001.zip › brainsci-3424594-supplementary.pdf]

Table S1. Correlation analysis for molecular and behavioral measures.

	Bdnf exon 4		***Bdnf exon 6***		***Bdnf exon 9***	
	HIP	CPF	HIP	ÇPF	HIP	CPF
**Open field test**<br>Crossings	r^2=0.139<br>F(1,15)=2.26<br>p = 0.155	r^2=0.096<br>F(1,15)=1.50<br>p = 0.242	r^2=0.003<br>F(1,15)=0.045<br>p = 0.834	r^2=0.064<br>F(1,15)=0.95<br>p = 0.345	r^2=0.109<br>F(1,15)=1.71<br>p = 0.212	r^2=0.107<br>F(1,15)=1.67<br>p = 0.217
Rearings	r^2=0.131<br>F(1,15)=2.11<br>p = 0.169	r^2=0.002<br>F(1,15)=0.04<br>p = 0.846	r^2=0.050<br>F(1,15)=0.59<br>p = 0.453	r^2=0.018<br>F(1,15)=0.25<br>p = 0.623	r^2=0.036<br>F(1,15)=0.52<br>p = 0.480	r^2=0.027<br>F(1,15)=0.39<br>p = 0.540
**Elevated plus maze**<br>Total entries	r^2=0.277<br>F(1,15)=5.36<br>p = 0.036	r^2=0.105<br>F(1,15)=1.63<br>p = 0.222	r^2=0.023<br>F(1,15)=0.43<br>p = 0.523	r^2=0.196<br>F(1,15)=3.41<br>p = 0.086	r^2=0.246<br>F(1,15)=4.61<br>p = 0.050	r^2=0.205<br>F(1,15)=3.60<br>p = 0.078
Rearings	r^2=0.074<br>F(1,15)=1.11<br>p = 0.310	r^2=0.015<br>F(1,15)=0.21<br>p = 0.652	r^2=0.003<br>F(1,15)=0.05<br>p = 0.824	r^2=0.082<br>F(1,15)=1.24<br>p = 0.284	r^2=0.054<br>F(1,15)=0.80<br>p = 0.386	r^2=0.134<br>F(1,15)=2.17<br>p = 0.163
Head dips	r^2=0.074<br>F(1,15)=1.14<br>p = 0.310	r^2=0.015<br>F(1,15)=0.10<br>p = 0.652	r^2=0.003<br>F(1,15)=0.10<br>p = 0.751	r^2=0.082<br>F(1,15)=0.16<br>p = 0.689	r^2=0.046<br>F(1,15)=0.67<br>p = 0.424	r^2=0.019<br>F(1,15)=0.27<br>p = 0.607

Open arm entries (n)	r^2=0.045<br>F(1,15)=0.67<br>p = 0.426	r^2=0.001<br>F(1,15)=<br>0.0000246<br>p = 0.996	r^2=0.025<br>F(1,15)=0.35<br>p = 0.562	r^2=0.010<br>F(1,15)=0.14<br>p = 0.709	r^2=0.030<br>F(1,15)=0.43<br>p = 0.520	r^2=0.012<br>F(1,15)=0.17<br>p = 0.683
Open arm duration (%)	r^2=0.037<br>F(1,15)=0.53<br>p = 0.480	r^2=0.012<br>F(1,15)=0.17<br>p = 0.683	r^2=0.045<br>F(1,15)=0.73<br>p = 0.406	r^2=0.004<br>F(1,15)=0.05<br>p = 0.818	r^2=0.001<br>F(1,15)=0.007<br>p = 0.934	r^2=0.008<br>F(1,15)=0.103<br>p = 0.754
Closed arm entries (n)	r^2=0.095<br>F(1,15)=1.47<br>p = 0.246	r^2=0.092<br>F(1,15)=1.42<br>p = 0.253	r^2=0.045<br>F(1,15)=0.66<br>p = 0.430	r^2=0.121<br>F(1,15)=1.92<br>p = 0.187	r^2=0.128<br>F(1,15)=2.05<br>p = 0.174	r^2=0.126<br>F(1,15)=2.01<br>p = 0.178
Closed arm duration (%)	r^2=0.143<br>F(1,15)=2.17<br>p = 0.165	r^2=0.014<br>F(1,15)=0.19<br>p = 0.667	r^2=0.053<br>F(1,15)=0.78<br>p = 0.391	r^2=0.003<br>F(1,15)=0.04<br>p = 0.833	r^2=0.0002<br>F(1,15)=0.003<br>p = 0.952	r^2=0.007<br>F(1,15)=0.09<br>p = 0.767
**Forced swimming test**<br>Swimming	**r^2=0.551**<br>**F(1,15)=17.15**<br>**p = 0.001**	**r^2=0.380**<br>**F(1,15)=8.57**<br>**p = 0.011**	r^2=0.108<br>F(1,15)=1.68<br>p = 0.215	**r^2=0.469**<br>**F(1,15)=12.37**<br>**p = 0.003**	**r^2=0.437**<br>**F(1,15)=10.88**<br>**p = 0.005**	**r^2=0.438**<br>**F(1,15)=10.89**<br>**p = 0.005**
Immobility	r^2=0.105<br>F(1,15)=1.63<br>p = 0.222	r^2=0.002<br>F(1,15)=0.025<br>p = 0.877	r^2=0.028<br>F(1,15)=0.39<br>p = 0.538	r^2=0.016<br>F(1,15)=0.23<br>p = 0.639	r^2=0.045<br>F(1,15)=0.65<br>p = 0.432	r^2=0.023<br>F(1,15)=0.32<br>p = 0.578

Climbing	r^2=0.010<br>F(1,15)=0.14<br>p = 0.712	r^2=0.046<br>F(1,15)=0.67<br>p = 0.426	r^2=0.191<br>F(1,15)=3.30<br>p = 0.091	r^2=0.007<br>F(1,15)=0.10<br>p = 0.754	r^2=0.0005<br>F(1,15)=0.008<br>p = 0.930	r^2=0.001<br>F(1,15)=0.01<br>p = 0.904

HIP = hippocampus; CPF = prefrontal cortex
